# Supplementary material for: The Requirement for US28 During Cytomegalovirus Latency Is Independent of US27 and US29 Gene Expression
Source: Front Cell Infect Microbiol. 2020 Apr 28;10:186. doi: 10.3389/fcimb.2020.00186 (PMC7198828; doi:10.3389/fcimb.2020.00186)
Supplement: Supplementary file 1 [file Data_Sheet_1.pdf]

Supplementary Material

The Requirement For US28 During Cytomegalovirus Latency Is Independent Of US27 And US29 Gene Expression

Benjamin A. Krishna[1§], Amanda B. Wass[1§], Rajashri Sridharan[1], Christine M. O'Connor[1*]

1 Supplementary Data

Supplementary Tables: 1

Supplementary Figures: 3

Supplementary Table 1. Oligonucleotides used in this study.

Primer Use	Primer Direction	Primer Sequence
**3xF-Kan-frt insertion**	forward[§]	TCTTCCGACACGCTGTCCGACGAGGTGTGTCGCGTCTCACAAATTATACCGTTAGATTATAAAGATGATGATGATAAA
	reverse[§]	AGAGGGGCGGACACGGGGTTTGTATGAAAAGGCCGAGGTAGCGCTTTTTTAGGCCGCGGGAATTCGAAGTT
***galK* insertion**	forward[¶]	GCACCGAGGGCAGAACTGGTGCTATCATGACACCGACGACGACGACCGCGcctgttgacaattaatcatcggca
	reverse[¶]	TGAAAACACAAGGAGTCGCGTCTTCATCGTAGTCAAACTCCGTCGTGAGTTtcagcactgtcctgctcctt
***galK* reversion oligo**	top	CACCGAGGGCAGAACTGGTGCTATCATGACACCGACGACGACGACCGCGTAACTCACGACGGAGTTTGACTACGATGAAGACGCGACTCCTTGTGTTTT
	bottom	AAAACACAAGGAGTCGCGTCTTCATCGTAGTCAAACTCCGTCGTGAGTTACGCGGTCGTCGTCGTCGGTGTCATGATAGCACCAGTTCTGCCCTCGGTG
**Multi-tag vGPCR virus – *galK* insertion**		
UL33-c-myc	forward[¶]	ACAAAAATCCCCCATCGACTCTCACAATCGCATCATAACCTCAGCGGGGTAcctgttgacaattaatcatcggca
	reverse[¶]	AAATGGCGACGGGTTCTGGTGCTTTCTGAATAAAGTAACAGGAAAGCTCAtcagcactgtcctgctcctt
UL78-V5	forward[¶]	TGCACCGACGGCGAAAACACCGTCGCGTCCGACGCAACGGTGACGGCATTAcctgttgacaattaatcatcggca
	reverse[¶]	GTGATTTATCTGCCACTTTTCTCCCCGCTGCCGTACAGCGCCGCCGCTCAtcagcactgtcctgctcctt
US27-3xHA	forward[¶]	TATGACAGAAAAAATGCACCTATGGAGTCCGGGGAGGAGGAATTTCTATTGcctgttgacaattaatcatcggca

	reverse¶	CAATGAGCAAAAATAGATGTGCGGCGGACGCGTGAAAGAGGATCGAATTAtcagcactgtcctgctcctt
**Multi-tag vGPCR virus – *galK* reversion gBlocks**		
UL33-c-myc		ACAAAAATCCCCCATCGACTCTCACAATCGCATCATAACCTCAGCGGGGTAGAACAAAAACTTATTTCTGAAGAAGATCTTTGAGCTTTCCTGTTACTTTATTCAGAAAGCACCAGAACCCGTCGCCATTT
UL78-V5		TGCACCGACGGCGAAAACACCGTCGCGTCCGACGCAACGGTGACGGCATTAGGTAAGCCTATCCCTAACCCTCTCCTCGGTCTCGATTCTACGTGAGCGGCGGCGCTGTACGGCAGCGGGGAGAAAAGTGGCAGATAAATCAC
US27-3xHA		TATGACAGAAAAAATGCACCTATGGAGTCCGGGGAGGAGGAATTTCTATTGTACCCATATGACGTTCCAGACTACGCGTATCCGTACGACGTTCCGGATTACGCTTACCCTTACGACGTACCTGACTACGCTTAATTCGATCCTCTTTCACGCGTCCGCCGCACATCTATTTTTGCTCATTG
**Multi-tag vGPCR virus – *galK* reversion gBlock amplifying primers**		
UL33-c-myc	forward	ACAAAAATCCCCCATCGACTC
	reverse	AAATGGCGACGGGTTCTGGTG
UL78-V5	forward	TGCACCGACGGCGAAAACACC
	reverse	GTGATTTATCTGCCACTTTTC
US27-3xHA	forward	TATGACAGAAAAAATGCACCT
	reverse	CAATGAGCAAAAATAGATGTG
**RTqPCR**		
US27	forward	CCGTATGGTGCGGTTTATCATTA
	reverse	CTAAAAATAGCGCCAGGTTGAAAGG
US28	forward	CCAGAATCGTTGCGGTGTCTCAGT
	reverse	CGTGTCCACAAACAGCGTCAGGT
US29	forward	CGACGAGACAACAATGAC

	reverse	AATTGACGGTCCACTGAG
UL123	forward	GCCTTCCCTAAGACCACCAAT
	reverse	ATTTTCTGGGCATAAGCCATAATC
UL99	forward	GTGTCCCATTCCCGACTCG
	reverse	TTCACAACGTCCACCCACC
GAPDH	forward	ACCCACTCCTCCACCTTTGAC
	reverse	CTGTTGCTGTAGCCAAATTCGT

§ Underlined sequences are complementary to the pKan-frt template.
¶ Lowercase sequences are complementary to the pGalK template.

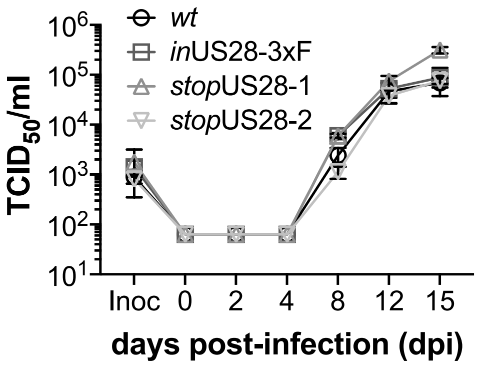

Supplementary Figure 1. US28 recombinant viruses display wild type growth kinetics in lytically-infected primary fibroblasts. MRC-5 cells were infected as indicated (moi = 0.01). Supernatants from each culture were collected at the indicated days post-infection (dpi). Infectious virus was quantified by $TCID_{50}$ on naïve fibroblasts. Inoc, inoculum. All samples were analyzed in triplicate. Error bars indicate standard deviation, and statistical significance was calculated using two-way ANOVA analyses followed by Tukey's post-hoc analyses. Values were not statistically significant.

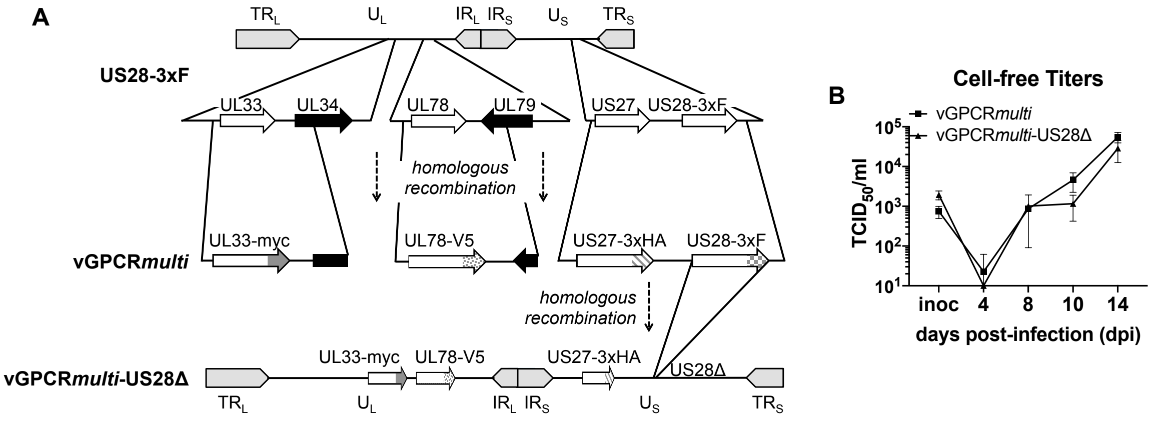

Supplementary Figure 2. TB40/E*mCherry*-vGPCR*multi* and- vGPCR*multi*-US28Δ replicate to wild type titers in lytically infected fibroblasts. (A) TB40/E*mCherry*-US28-3xF (Miller et al., 2012) was used to generate TB40/E*mCherry*-vGPCR*multi* (vGPCR*multi*), containing epitope tags on each additional vGPCR, as depicted. vGPCR*multi* was subsequently used to generate vGPCR*multi*-US28Δ, which lacks the entire US28 ORF, inclusive of the triple FLAG epitope tag. **(B)** Multi-step growth analyses of the viral recombinants in **(A)** was evaluated in lytically infected NuFF-1 fibroblasts (moi = 0.01). At the indicated times post-infection, infectious supernatants were collected, and titers were determined using $TCID_{50}$ assay on naïve NuFF-1 fibroblasts in triplicate. Inoc, inoculum. Error bars indicate standard deviation, and statistical significance was calculated using two-way ANOVA with Sidak's post-hoc analysis. Values were not statistically significant.

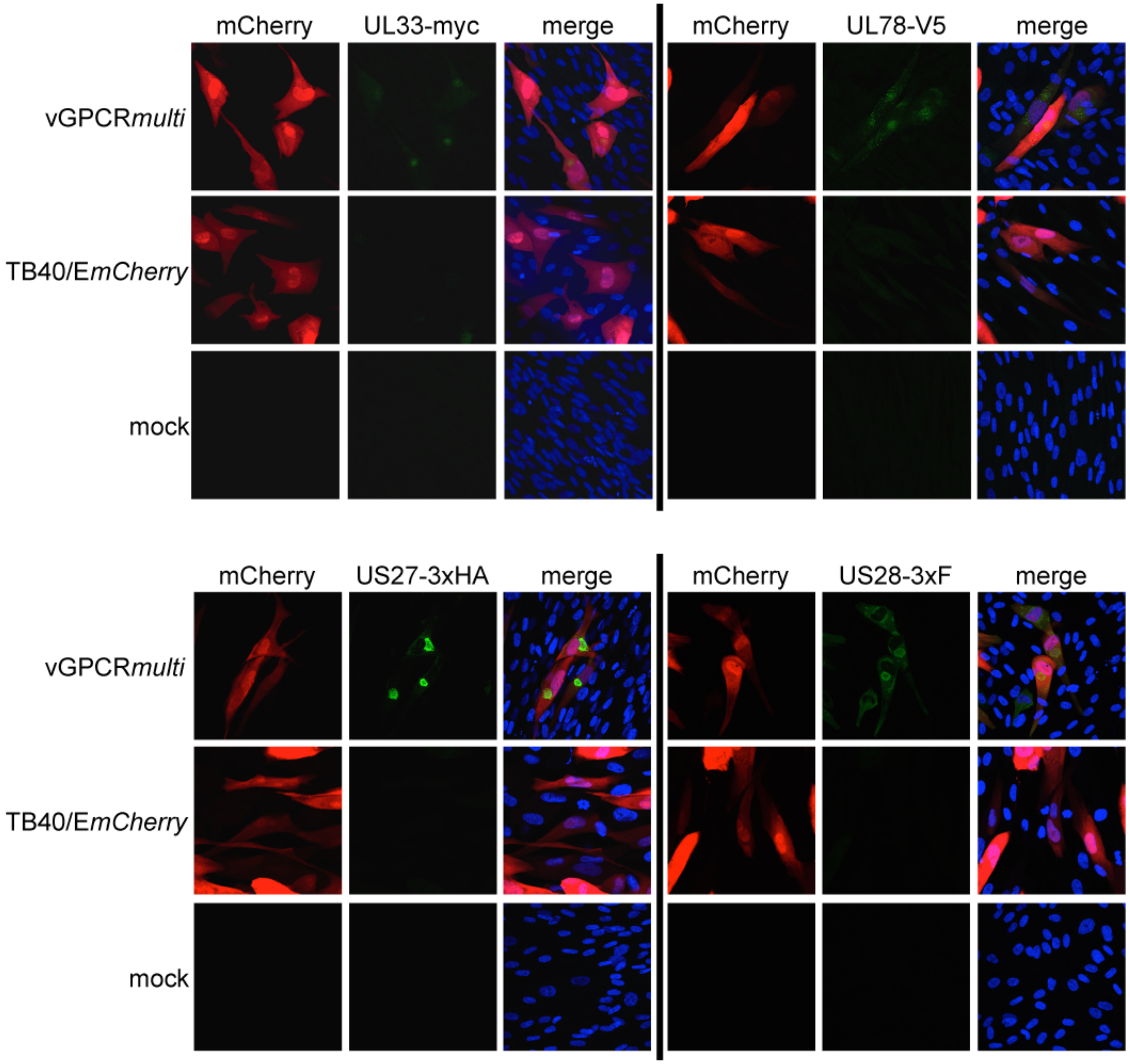

Supplementary Figure 3. TB40/E*mCherry*-vGPCR*multi* expresses each vGPCR following lytic infection. NuFF-1 fibroblasts were mock-infected or lytically infected (moi = 0.5) as shown and harvested 96 hpi for IFA. The appropriate antibodies were used to detect the indicated epitope tag (see Materials and Methods for specific antibody details): UL33 (anti-myc), UL78 (anti-V5), US27 (anti-HA), US28 (anti-FLAG), each shown in green. mCherry (red) is a marker of infection, and nuclei were visualized with DAPI (blue). Images were acquired with a 40x objective with 1.5x magnification. Representative images are shown (n=3).
